# Neuroendocrine Transformation Without PSA Elevation in High‐Risk Metastatic Castration‐Sensitive Prostate Cancer Under Androgen Receptor Signaling Inhibition: A Case Report

**DOI:** 10.1002/iju5.70196

**Published:** 2026-05-27

**Authors:** Yoshihiro Nakagami, Shintaro Ohnuma, Masashi Tamaoka, Tatsuki Inoue, Motoki Yamagishi, Kazuhiko Oshinomi, Masakazu Nagata, Yasuyuki Oohira, Toshiko Yamochi, Takashi Fukagai

**Affiliations:** ^1^ Department of Urology Showa Medical University School of Medicine Tokyo Japan; ^2^ Department of Pathology Showa Medical University School of Medicine Tokyo Japan

**Keywords:** abiraterone acetate, amphicrine prostate cancer, androgen receptor signaling inhibitor, metastatic castration‐sensitive prostate cancer, neuroendocrine prostate cancer

## Abstract

**Introduction:**

Neuroendocrine prostate cancer is a rare and highly aggressive variant of prostate cancer with a poor prognosis. We report a case in which metastatic castration‐sensitive prostate cancer directly transformed into neuroendocrine prostate cancer without a corresponding increase in prostate‐specific antigen.

**Case Presentation:**

An 86‐year‐old male with severe acute renal failure demonstrated a prostate‐specific antigen level of 306 ng/mL and a Gleason score of 4 + 5 on prostate biopsy. Imaging revealed lung, bone, and lymph node metastases, leading to a diagnosis of high‐risk metastatic castration‐sensitive prostate cancer. Abiraterone acetate was initiated immediately. Nineteen months later, multiple liver metastases were detected despite stable prostate‐specific antigen levels. The patient died 4 months later. Autopsy revealed widespread metastases, all histologically confirmed as neuroendocrine prostate cancer.

**Conclusion:**

Strong androgen receptor suppression in metastatic castration‐sensitive prostate cancer can lead to aggressive radiographic progression without prostate‐specific antigen elevation, underscoring the limitations of antigen‐based monitoring.

## Keynote Message

This case highlights that aggressive prostate cancer can progress silently on imaging without an accompanying rise in prostate‐specific antigen, particularly during treatment with potent androgen receptor signaling inhibitors. Reliance on prostate‐specific antigen alone may delay recognition of highly aggressive disease transformation, such as neuroendocrine prostate cancer. Our findings emphasize the importance of regular imaging and clinical vigilance, even when blood markers appear controlled. Early identification of discordant disease progression may allow timely diagnostic biopsy and more appropriate treatment decisions, ultimately improving patient outcomes.

**Abbreviations**
ADT: androgen deprivation therapy
AMPC: amphicrine prostate cancer
AR: androgen receptor
ARSI: androgen receptor signaling inhibitor
AUA: American Urological Association
CRPC: castration‐resistant prostate cancer
CSC: cancer stem cell
CSPC: castration‐sensitive prostate cancer
EAU: European Association of Urology
INSM1: insulinoma‐associated protein 1
mCSPC: metastatic castration‐sensitive prostate cancer
NEPC: neuroendocrine prostate cancer
NSE: neuron‐specific enolase
OS: overall survival
PD: progressive disease
Pro‐GRP: pro–gastrin‐releasing peptide
PSA: prostate‐specific antigen
RB1: retinoblastoma 1
RRT: renal replacement therapy
SEER: surveillance, epidemiology and end results database
t‐NEPC: treatment‐related neuroendocrine prostate cancer

## 1 Introduction

Neuroendocrine prostate cancer (NEPC) is an extremely rare and aggressive variant of prostate cancer associated with a poor prognosis. It may present with metastasis, especially to the central nervous system. Herein, we report a rare case in which high‐risk metastatic castration‐sensitive prostate cancer (mCSPC) transformed directly into NEPC without prostate‐specific antigen (PSA) elevation. Disease progression was detected radiographically, and pathological autopsy confirmed NEPC of the primary prostate cancer and all metastatic lesions.

## 2 Case Presentation

**FIGURE 1:**
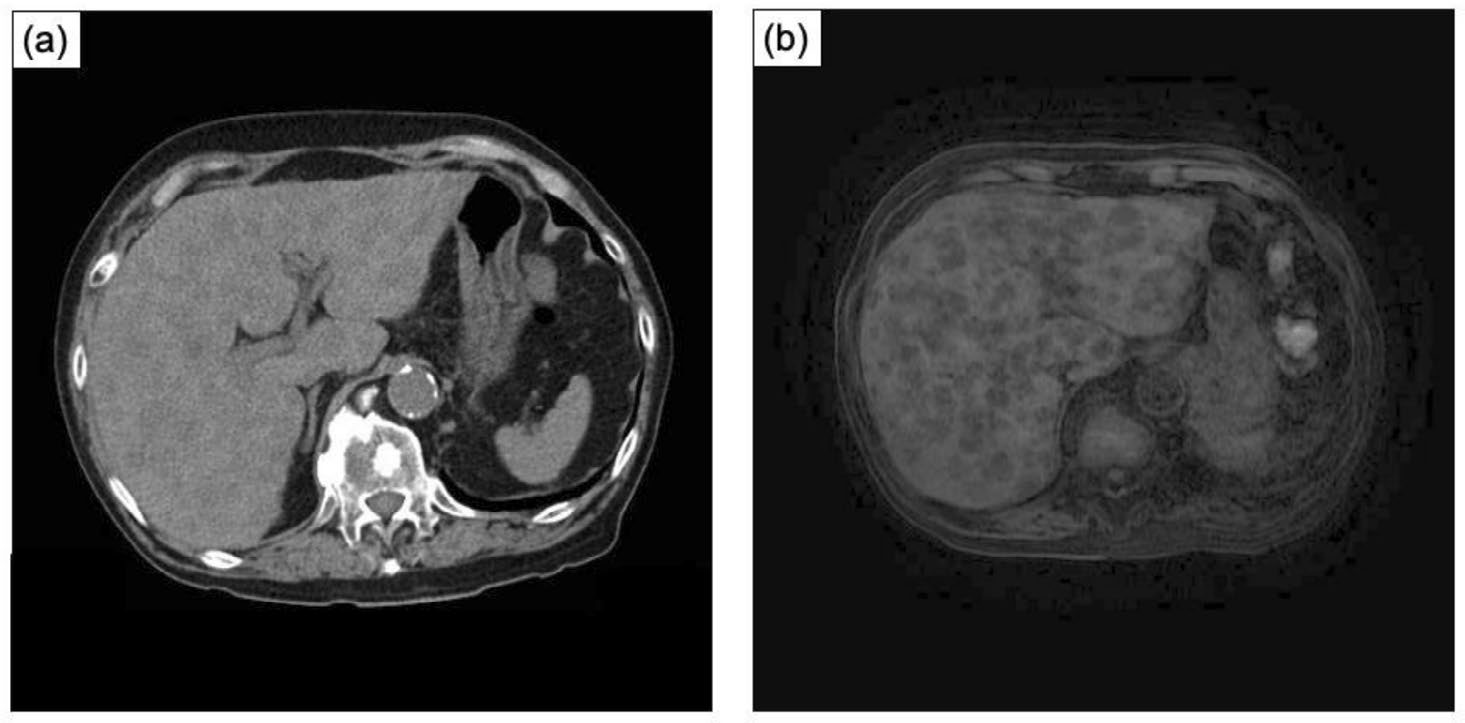
Plain computed tomography (a) and plain magnetic resonance image (b) scans showing multiple liver metastasis.

**FIGURE 2:**
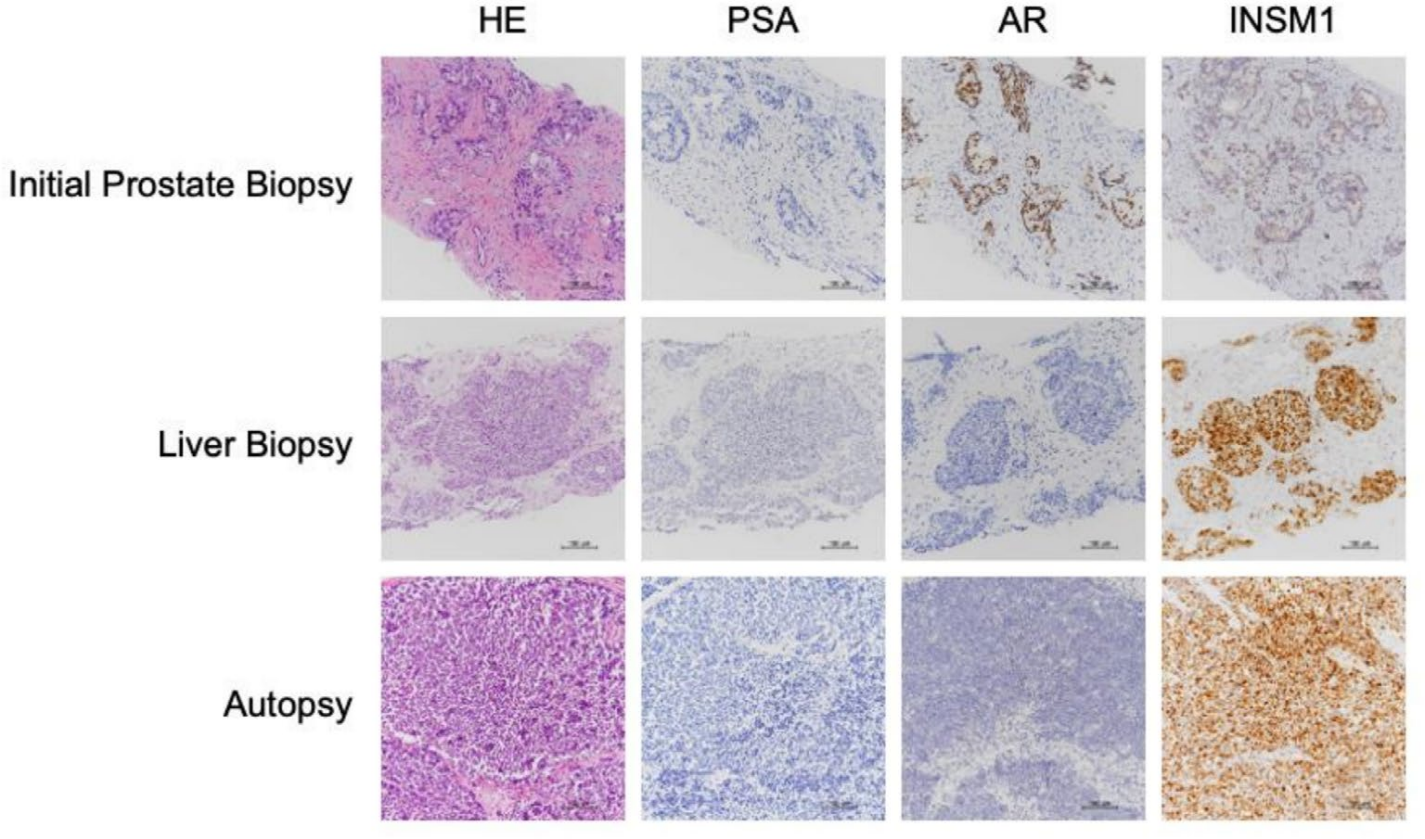
Pathological specimens including immunostaining at initial prostate biopsy, liver biopsy, and autopsy. Prostate‐specific antigen immunostaining was negative in all cases. Androgen receptor immunostaining was positive only in initial prostate biopsy specimens. A small amount of weakly positive immunostaining for insulinoma‐associated protein 1 (INSM1) is present in the initial prostate biopsy, with strongly positive INSM1 staining in the liver biopsy and autopsy specimens. AR, androgen receptor; HE, hematoxylin and eosin; INSM1, insulinoma‐associated protein 1; PSA, prostate‐specific antigen.

**FIGURE 3:**
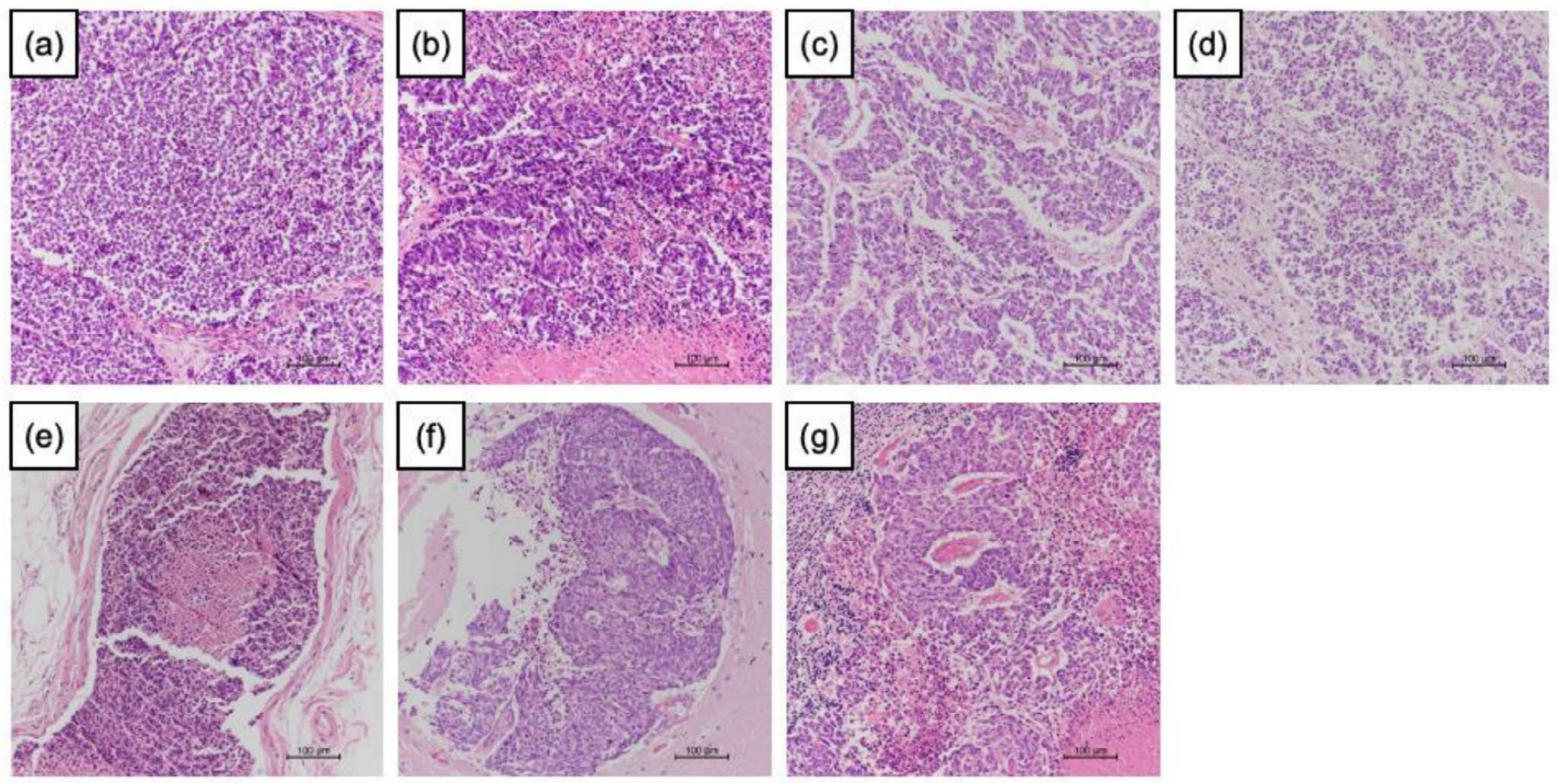
Pathological specimens at autopsy. Neuroendocrine prostate cancer was diagnosed in primary prostate tumor (a), lungs (b), liver (c), left adrenal gland (d), gallbladder (e), cerebrum (f), and cerebellum (g) with hematoxylin and eosin staining.

An 86‐year‐old male initially presented with severe acute renal failure. The initial PSA level was 306 ng/mL. Prostate biopsy revealed a Gleason score of 4 + 5 was in all 12 cores. Imaging revealed lung, bone, and lymph node metastases, leading to a diagnosis of high‐risk CSPC. Treatment with luteinizing hormone‐releasing hormone antagonist and abiraterone acetate was initiated immediately. Fourteen months later, radiographic findings improved, with a PSA nadir of 0.465 ng/mL. After 19 months of treatment, the patient developed anorexia and fatigue. Laboratory testing revealed liver dysfunction, with an aspartate aminotransferase level of 110 U/L (normal range: 13–30 U/L), alanine aminotransferase level of 80 U/L (10–42 U/L), γ‐glutamyl transpeptidase level of 276 U/L (13–64 U/L), lactate dehydrogenase level of 1855 U/L (120–220 U/L), and alkaline phosphatase level of 269 U/L (38–113 U/L). Radiographic examinations revealed multiple liver metastases. (Figure 1) The PSA level was 1.398 ng/mL, and did not meet criteria for biochemical recurrence. In contrast, neuroendocrine markers were markedly elevated, including pro‐gastrin‐releasing peptide (ProGRP) (545 pg/mL, normal range < 81.0 pg/L) and neuron‐specific enolase (NSE) (746 ng/mL, normal range < 16.3 ng/mL). Given the patient's advanced age and poor general condition, a clinical diagnosis of NEPC was made without liver biopsy. Cisplatin‐based chemotherapy was not feasible due to renal dysfunction, and four courses of docetaxel monotherapy were administered. Following partial improvement in general condition, liver biopsy was performed, which confirmed NEPC with insulinoma‐associated protein 1 (INSM1) positivity (Figure 2) and breast cancer susceptibility gene negativity. Neuroendocrine markers were dropped to nadia including ProGRP (119.3 pg/mL) and NSE (132.0 ng/mL) 2 months after the start of chemotherapy. Despite treatment, the disease progressed rapidly. Rapid increasing of ProGRP and NSE was recorginized including ProGRP (953.4 pg/mL) and NSE (2290.0 ng/mL), and the patient died 4 months after the onset of liver metastases. Pathological autopsy revealed metastases involving both lungs, liver, left adrenal gland, gallbladder, cerebrum, and cerebellum. (Figure 3) All metastatic lesions, including the primary prostate tumor, were pathologically diagnosed as NEPC. No metastatic lesions were found in esophagus, heart, aorta, intestinal tract and kidney. Retrospective review of pathological specimens from the initial prostate biopsy (Figure 2) demonstrated a small neuroendocrine component, which had expanded markedly across all organs. Immunohistochemistry showed negative PSA and androgen receptor (AR) staining.

## 3 Discussion

According to the Surveillance, Epidemiology, and End Results (SEER) database, NEPC accounted for 476 of 780 379 prostate cancer cases diagnosed between 2004 and 2017 [1]. NEPC is broadly classified into de novo and treatment‐related NEPC (t‐NEPC). The incidence of NEPC has increased in the era of next‐generation AR signaling inhibitors (ARSIs) and t‐NEPC is estimated to occur in 17%–30% of castration‐resistant prostate cancers (CRPC) cases [2]. In contrast, de novo NEPC comprises < 2% of organ‐confined prostate cancer [3].

At the molecular level, NEPC generally shows few genomic changes aside from *MYCN* amplification, *TP53* mutation or deletion, and *RB1* loss. This indicates that trans‐differentiation is primarily driven by epigenetic factors and/or tumor microenvironment signals [4]. *RB1* and *TP53* loss occur in 70%–90% and 56%–67% of cases [5, 6], respectively, and their co‐occurrence correlates with lower overall survival after NEPC diagnosis [4]. While NEPC's aggressive growth is linked to p53 and Rb1 pathway disruption, NE marker overexpression may result from other mechanisms [7]. These data suggest that epithelial plasticity—mediated by alterations in Rb1, p53, PTEN, N‐Myc, and increased EZH2 and SOX2—produces heterogeneous AR‐indifferent cells, with further changes like those in SRRM4‐REST driving terminal NEPC differentiation [8].

Two principal mechanisms have been proposed to explain neuroendocrine differentiation in response to ARSIs [6]. In the hierarchical model, as tumors are heterogeneous, ARSIs can reduce tumor burden except neuroendocrine cell and cancer stem‐like cells (CSCs). The population of tumorigenic CSCs expands after ARSIs treatment, and CSCs differentiate into malignant neuroendocrine cells. In the dynamic trans‐differentiation model, luminal epithelial cells can be reprogrammed into neuroendocrine cells under ARSI pressure, leading to partial epithelial‐mesenchymal transition, which generates CSCs.

Cisplatin‐based chemotherapy remains the primary treatment for NEPC, while emerging therapeutic strategies include inhibitors targeting interleukin‐6–signal transducer and activator of transcription 3 signaling, cancer stem cells, MYCN, aurora kinase A, and enhancer of zeste homolog 2 [9].

In the present case, imaging showed disease progression without increased PSA levels. Definitions of biochemical recurrence in mCSPC vary slightly among guidelines, including those of the European Association of Urology [10] and the American Urological Association [11]. This case did not meet biochemical recurrence criteria under either definition. Notably, the CHAARTED trial demonstrated clinical progression without PSA elevation in a substantial proportion of patients receiving androgen deprivation therapy and was associated with worse overall survival [12]. Similarly, Hara et al. showed that discordance between PSA levels and imaging findings can occur at any disease stage [13], underscoring the importance of imaging‐based surveillance regardless of biochemical response. According to the NCCN guidelines, follow‐up for mCSPC includes physical examination and PSA assessment every 3–6 months, imaging when clinically indicated, and periodic imaging to evaluate treatment response. If significant metastatic progression is detected on imaging despite low PSA levels, NEPC should be suspected, and metastatic biopsy and assessment of neuroendocrine markers should be considered.

T‐NEPC typically arises in patients with CRPC. To our knowledge, only one prior report has described NEPC without PSA elevation while using abiraterone acetate for mCSPC [14]. In our case, retrospective pathological evaluation revealed a small neuroendocrine component in the initial biopsy and positive AR staining, suggesting an amphicrine phenotype. Amphicrine prostate cancer (AMPC), characterized by co‐expression of AR and neuroendocrine markers, has been proposed [15], with significantly longer survival compared with NEPC. No patients with AMPC exhibit RB1 loss (a characteristic of NEPC). Thus, loss of neural transcriptional repressors is associated with the development of both NEPC and AMPC.

## 4 Conclusion

This case demonstrates that strong AR suppression in mCSPC can lead to aggressive imaging progression without matching the definition of CRPC and a direct transformation to t‐NEPC. In the era of ARSIs, recurrence cannot be diagnosed using PSA levels alone. Regular imaging and consideration of metastatic biopsies are essential with NEPC. In patients with highly aggressive tumors such as high‐risk mCSPC, these examinations are necessary to identify NEPC in biopsy specimens.

## Ethics Statement

This case report was approved by the Ethics Committee of Showa Medical University Hospital (Study No. CR2024041‐A).

## Consent

Written informed consent was obtained from the patient for publication of this article.

## Conflicts of Interest

The authors declare no conflicts of interest.

## Data Availability

The data that support the findings of this study are available on request from the corresponding author. The data are not publicly available due to privacy or ethical restrictions.
